# Body mass index, as a novel predictor of hepatocellular carcinoma patients treated with Anti-PD-1 immunotherapy

**DOI:** 10.3389/fmed.2022.981001

**Published:** 2022-09-20

**Authors:** Jierong Chen, Lianghe Lu, Chunhua Qu, Gari A, Fangqi Deng, Muyan Cai, Wei Chen, Lie Zheng, Jiewei Chen

**Affiliations:** ^1^State Key Laboratory of Oncology in South China, Collaborative Innovation Center for Cancer Medicine, Sun Yat-sen University Cancer Center, Guangzhou, China; ^2^Department of Pathology, Sun Yat-sen University Cancer Center, Guangzhou, China; ^3^Department of Hepatobiliary Oncology, Sun Yat-sen University Cancer Center, Guangzhou, China; ^4^Department of Pancreatobiliary Surgery, Center of Hepato-Pancreato-Biliary Surgery, The First Affiliated Hospital of Sun Yat-sen University, Guangzhou, China; ^5^Department of Imaging, Sun Yat-sen University Cancer Center, Guangzhou, China

**Keywords:** hepatocellular carcinoma, body mass index, anti-PD-1 antibody, progression-free survival, immune-related adverse events

## Abstract

Immunocheckpoint inhibitors have shown significant efficacy in the treatment of hepatocellular carcinoma (HCC), but there are individual differences. The aim of this study was to explore body mass index (BMI) as a predictor of anti-PD-1 efficacy in patients with HCC. We retrospectively analyzed 101 HCC patients who treated with anti-PD-1 at Sun Yat-sen University Cancer Center from July 2018 to November 2019 and divided them into overweight (BMI > 24.9) and non-overweight (BMI ≤ 24.9) groups based on baseline BMI levels. BMI > 24.9 accounted for 22 cases (21.8%) and BMI ≤ 24.9 accounted for 79 cases (78.2%) in the study cohort. Overweight patients had higher disease control rates than non-overweight patients (*P* = 0.019, respectively). The mean progression-free survival (PFS) in overweight patients (10.23 months) was significantly longer than that of non-overweight patients (6.85 months; *P* = 0.027). Among patients with immune-related adverse events (irAEs), the mean PFS was also significantly longer in overweight patients (7.72 months) than in non-overweight patients (5.31 months, *P* = 0.034). Multivariate analysis showed that BMI was an independent prognostic factor for PFS in HCC patients treated with anti-PD-1 (hazard ratio: 0.47, *P* = 0.044). Thus, higher BMI predicts a better prognosis among HCC patients treated with anti-PD-1. In clinical practice, patients' BMI can provide a useful tool for predicting the efficacy of anti-PD-1 therapy.

## Introduction

Hepatocellular carcinoma (HCC) is the most common primary liver cancer. According to the 2020 global cancer statistics, HCC has the sixth highest incidence and the third highest death rate (1, 2). HCC is one of the most common refractory cancers worldwide.

Most HCC patients are diagnosed at advanced stage, and radical therapy is not applicable (1). Targeted drugs such as sorafenib have unsatisfactory therapeutic effects and can only prolong the overall survival of patients by several months. Drug resistance and adverse reactions limit the continued use of drugs (3). With the development of immunotherapy, immune checkpoint inhibitors (ICIs) have shown significant efficacy in the treatment of HCC (4–6). The strong immune-mediated pathogenesis of HCC makes this cancer a remarkable anticancer response to immune-based therapy (7). Programmed cell death receptor-1 (PD-1) is a classical immune checkpoint, and anti-PD-1 antibodies play an important role in the treatment of HCC (8). However, there are significant individual differences in the efficacy of anti-PD-1 antibodies, and it has become a hot topic of clinical research in recent years to explore which index patients can benefit more from anti-PD-1 antibody therapy.

Adipose tissue may affect the response of cancer patients to ICIs. Overweight is a tumorigenic immune dysfunction that can be reversed by ICIs (9). The body mass index (BMI), as a major indicator of body weight and nutritional status, is correlated with efficacy of many drugs (10). BMI is a useful predictor of prognosis in patients with renal cell carcinoma treated with targeted therapy (11). In metastatic melanoma, BMI may predict tumor response to ICIs (12). However, the effect of BMI on tumor response in patients with HCC receiving anti-PD-1 therapy remains unclear.

To further clarify these questions, we retrospectively analyzed HCC patients treated with anti-PD-1 antibodies and assessed progression-free survival (PFS) based on baseline BMI to determine the potential impact of overweight and non-overweight status.

## Materials and methods

### Patients

A total of 101 patients diagnosed with HCC and treated with anti-PD-1 antibody at the Sun Yat-sen University Cancer Center from July 2018 to November 2019 were enrolled in our study. All patients received anti-PD-1 therapy, and had detailed medical history, clinical stage, treatment plan, radiological information, follow-up records and other clinical data. Of these patients, 3 (3.0%) were treated with nivolumab, 9 (8.9%) were treated with pembrolizumab, 12 (11.9%) were treated with sintilimab, and 77 were treated (76.2%) with toripalimab. Nivolumab was administered at 1–3 mg/kg body weight every 2 weeks. Pembrolizumab was delivered intravenously at a fixed dose of 200 mg every 3 weeks. Toripalimab and sintilimab were given at 240 mg every 2 weeks and 200 mg every 3 weeks, respectively. From the start of anti-PD-1 therapy until 30 March 2020 all patients were followed up regularly with CT or MRI approximately every 3 months.

### Anthropometric measurements

BMI was calculated using a weight/height^2^ (kg/m^2^) formula based on the patients' documented heights and weights before the initiation of immunotherapy. Classification was performed according to WHO standards, and patients were classified as overweight (BMI > 24.9) and non-overweight (BMI ≤ 24.9) for final analysis using binomial cut-off values for BMI ≤ /> 24.9 (13). At the same time, immune-related adverse events (irAEs) recorded in patients were counted. IrAEs refers to side effects caused by immunodrug therapy, including allergic reactions, inflammation, diarrhea and other adverse reactions (14).

### Statistical analyses

Statistical analyses were performed using IBM SPSS Statistics version 26 (SPSS Inc., Chicago, IL, USA). PFS was calculated monthly from the first dose of anti-PD-1 antibody to progression or death, whichever occurred first, and the endpoint of progression was the time of first tumor enlargement, recurrence, emergence of new lesions or metastasis. The chi-square test was used to determine the relationship between BMI and tumor response. PFS curves were analyzed using the Kaplan-Meier method and compared using the logarithmic rank test. Cox multivariate regression analysis was used to determine risk factors for PFS. Statistical significance was set at *P* < 0.05.

## Results

### Baseline characteristics

A total of 101 patients were included in this study, including 82 males (81.2%) and 19 females (18.8%), with a median age of 50 years. Child-pugh grade A was found in 90 cases (89.1%) and child-pugh grade B in 11 cases (10.9%). Barcelona Clinic Liver Cancer (BCLC) stage A had 7 patients (6.9%), BCLC stage B had 10 patients (9.9%), and BCLC Stage C had 84 patients (83.2%). Before anti-PD-1 therapy, 41 patients received curative treatment (40.6%), 82 patients received local-regional therapy (81.2%), and 69 patients received target therapy (68.3%).

There were 22 (21.8%) patients with BMI > 24.9 (defined as overweight) and 79 (78.2%) patients with BMI ≤ 24.9 (defined as non-overweight). The chi-square test showed that the BMI of HCCs was significantly correlated with clinicopathological features, such as alpha-fetoprotein (AFP) level (*P* = 0.046), aspartate aminotransferase (AST) level (*P* = 0.000), creatinine (CRE) level (*P* = 0.014), and macroscopic vascular invasion (*P* = 0.037). However, no significant correlation was found with other clinicopathological features, such as Child-Pugh grade, Barcelona Clinic Liver Cancer (BCLC) stage, and prior treatment (*P* > 0.05). Detailed data are shown in Table 1.

**Table 1:** Correlations between body mass index and clinicopathological characteristics of hepatocellular carcinoma patients treated with anti- PD-1.

	**BMI > 24.9**	**BMI ≤ 24.9**	***P* value**
	**(*n* = 22)**	**(*n* = 79)**	
**Age (year)**			0.309
≤ 50	9 (40.9)	42 (53.2)	
>50	13 (59.1)	37 (46.8)	
**Gender**			0.187
Male	20 (90.9)	62 (78.5)	
Female	2 (9.1)	17 (21.5)	
**Hepatitis B infection**			0.280
Yes	21 (95.5)	69 (87.3)	
No	1 (4.5)	10 (12.7)	
**AFP (ng/ml)**			**0.046**
≤ 200	13 (59.1)	28 (35.4)	
>200	9 (40.9)	51 (64.6)	
**AST (u/l)**			**0.000**
≤ 53.5	19 (86.4)	32 (40.5)	
>53.5	3 (13.6)	47 (59.5)	
**CRE (umol/l)**			**0.014**
≤ 64.8	6 (27.3)	45 (57.0)	
>64.8	16 (72.7)	34 (43.0)	
**Tumor size (cm)**			0.522
≤ 5	12 (54.5)	37 (46.8)	
>5	10 (45.5)	42 (53.2)	
**Tumor number**			0.217
≤ 1	6 (27.3)	33 (41.8)	
>1	16 (72.7)	46 (58.2)	
**Macroscopic vascular invasion**			**0.037**
Present	15 (68.2)	34 (43.0)	
Absent	7 (31.8)	45 (57.0)	
**Extrahepatic metastasis**			0.293
Present	15 (68.2)	44 (55.7)	
Absent	7 (31.8)	35 (44.3)	
**irAEs**			0.350
Present	3 (13.6)	18 (22.8)	
Absent	19 (86.4)	61 (77.2)	
**Child-Pugh grade**			0.280
A	21 (95.5)	69 (87.3)	
B	1 (4.5)	10 (12.7)	
**ECOG PS**			0.459
0	7 (31.8)	32 (40.5)	
≥1	15 (68.2)	47 (59.5)	
**BCLC stage**			0.086
A	0 (0.0)	7 (8.9)	
B	1 (4.5)	9 (11.4)	
C	21 (95.5)	63 (79.7)	
**Prior treatment**			0.948
Curative treatment (Surgery, Ablation)	10 (45.5)	31 (39.2)	
Local-regional (TACE, HAIC, radiation)	19 (86.4)	63 (79.7)	
Target therapy (Sorafenib, lenvatinib)	15 (68.2)	54 (68.4)	

Variables are expressed as n (%).

BMI, body mass index; AFP, α-fetoprotein; AST, Aspartate Transaminase; CRE, Creatinine; irAEs: immune-related adverse events; ECOG PS, Eastern Cooperative Oncology Group performance status; BCLC stage, Barcelona Clinic Liver Cancer stage; TACE, transcatheter arterial chemoembolization; HAIC, hepatic arterial infusion of chemotherapy. Bold values indicate statistical significance.

### Association between BMI and antitumour response

According to RECIST 1.1 (curative effect evaluation standard of solid tumor), in overweight patients, one (4.5%) was rated as complete response (CR), three (13.6%) as partial response (PR), 15 (68.2%) as stable disease (SD), and three (13.6%) as progressive disease (PD). Among the non-overweight patients, seven (8.9%) had PR, 40 (50.6%) had SD, and 32 (40.5%) had PD. The overall response rate (ORR) was 18.2% (4/22) in overweight patients and 8.9% (7/79) in non-overweight patients (P=0.215). The disease control rate (DCR) was 86.4% (19/22) in overweight patients and 59.5% (47/79) in non-overweight patients (*P* = 0.019). The ORR and DCR of overweight individuals were higher than those of non-overweight individuals, and the DCR was more significant (Table 2). In addition, in the overweight group, there was only one complete response. After anti-PD-1 immunotherapy, there were no signs of active tumor in the liver. AFP decreased from 72 before treatment to 5.1, indicating a significant therapeutic effect.

**Table 2:** The relationship between tumor response and occurrence of BMI in HCC patients treated with anti-PD-1 antibodies.

**Tumor response**	**BMI > 24.9**	**BMI ≤ 24.9**	***p***
	**(*n* = 22)**	**(*n* = 79)**	
**CR**	1 (4.5)	0 (0.0)	
**PR**	3 (13.6)	7 (8.9)	
**SD**	15 (68.2)	40 (50.6)	
**PD**	3 (13.6)	32 (40.5)	
**ORR (CR+PR)**	4 (18.2)	7 (8.9)	0.215
**DCR (CR+PR+SD)**	19 (86.4)	47 (59.5)	**0.019**

BMI, body mass index; CR, complete response; PR, partial response; SD, stable disease; PD, progressive disease; ORR, overall response rate; DCR, disease control rate. Bold values indicate statistical significance.

### The risk factors for progression-free survival

**Figure 1:**
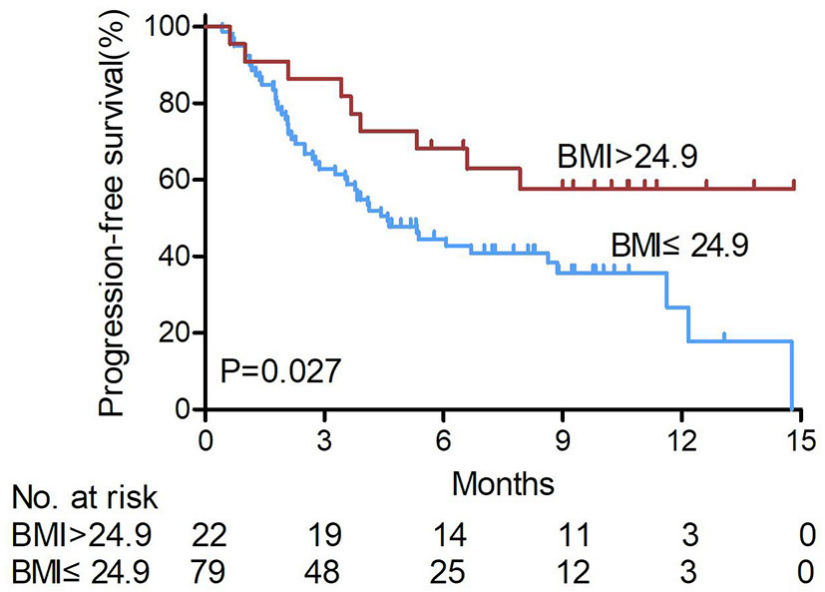
Kaplan-Meier survival curves of patients treated with anti-PD1 for hepatocellular carcinoma according to BMI levels. The mean PFS of overweight patients was significantly higher than that of non-overweight patients (log-rank test). BMI, body mass index. non-over weight BMI ≤ 24.9, over weight BMI > 24.9.

Kaplan-Meier survival analysis showed significant differences in BMI, AFP level, and irAEs (*P* = 0.027, *P* < 0.001, and *P* < 0.001, respectively; Table 3). There were risk factors for PFS in patients. The mean PFS of overweight patients was 10.23 months, which was significantly higher than that of non-overweight patients (6.85 months) (shown in Figure 1). The mean PFS of the patients with irAEs (irAEs+) was 12.11 months, which was significantly higher than that of the patients without irAEs (irAEs-) (6 months).

**Table 3:** Univariate analyses of risk factors for progression-free survival of hepatocellular carcinoma patients treated with anti- PD-1.

**Variable**	**All cases**	**Mean survival (months)**	**Chi-square value**	***p* value**
**Age, y**			1.689	0.194
≤ 50	51	6.99		
>50	50	8.49		
**Gender**			1.325	0.250
Female	19	6.63		
Male	82	8.22		
**Etiology**			0.314	0.575
Other	11	9.34		
Viral hepatitis	90	7.51		
**BMI**			4.895	**0.027**
≤ 24.9	79	6.85		
>24.9	22	10.23		
**AFP, ng/ml**			12.922	**0.000**
≤ 200	41	10.54		
>200	60	5.95		
**AST**			1.213	0.271
≤ 53.5	51	8.54		
>53.5	50	6.59		
**CRE**			0.809	0.368
≤ 64.8	51	7.20		
>64.8	50	8.34		
**Tumor size, cm**			0.209	0.648
≤ 5	49	8.16		
>5	52	6.99		
**Tumor number**			0.912	0.340
Single	39	7.72		
Multiple	62	7.23		
**Macrovascular invasion**			0.379	0.538
–	52	7.82		
+	49	7.34		
**Extrahepatic metastasis**			1.795	0.180
–	42	8.22		
+	59	7.30		
**irAEs**			13.018	**0.000**
–	80	6.00		
+	21	12.11		
**Child-Pugh grade**			1.432	0.231
A	90	7.98		
B	11	5.96		
**ECOG PS**			0.183	0.668
0	39	7.25		
≥1	62	7.82		

BMI, body mass index; AFP, α-fetoprotein; AST, Aspartate Transaminase; CRE, Creatinine; irAEs: immune-related adverse events; ECOG PS, Eastern Cooperative Oncology Group performance status. Bold values indicate statistical significance.

Further incorporating clinically significant factors into multivariate analysis, BMI (HR: 0.47, 95% CI: 0.22–0.98; *P* = 0.044) remained an independent risk and prognostic factor for PFS. AFP (HR: 2.42, 95% CI: 1.30–4.51; *P* = 0.005) and irAEs (HR: 0.18, 95% CI: 0.07–0.47; *P* < 0.001; Table 4) were also independent prognostic factors that significantly affected patients' PFS in this study cohort.

**Table 4:** Multivariate analyses of risk factors for progression-free survival of hepatocellular carcinoma patients treated with anti- PD-1.

	**HR (95% CI)**	***p* value**
**BMI** (>24.9 vs. ≤ 24.9)	0.47(0.22–0.98)	**0.044**
**AFP, ng/ml** (>200 vs. ≤ 200)	2.42(1.30–4.51)	**0.005**
**Tumor size, cm** (>5 vs. ≤ 5)	0.66(0.38–1.14)	0.133
**irAEs** (+ vs. –)	0.18(0.07–0.47)	**0.000**

HR, hazard ratio; CI, confidence interval; BMI, body mass index; AFP, α-fetoprotein; irAEs, immune-related adverse events. Bold values indicate statistical significance.

### Association between BMI and irAEs

Using the occurrence of irAEs as a stratification factor, the effect of BMI on the PFS of HCC patients receiving anti-PD-1 treatment was compared between irAEs- and irAEs+ patients.

**Figure 2:**
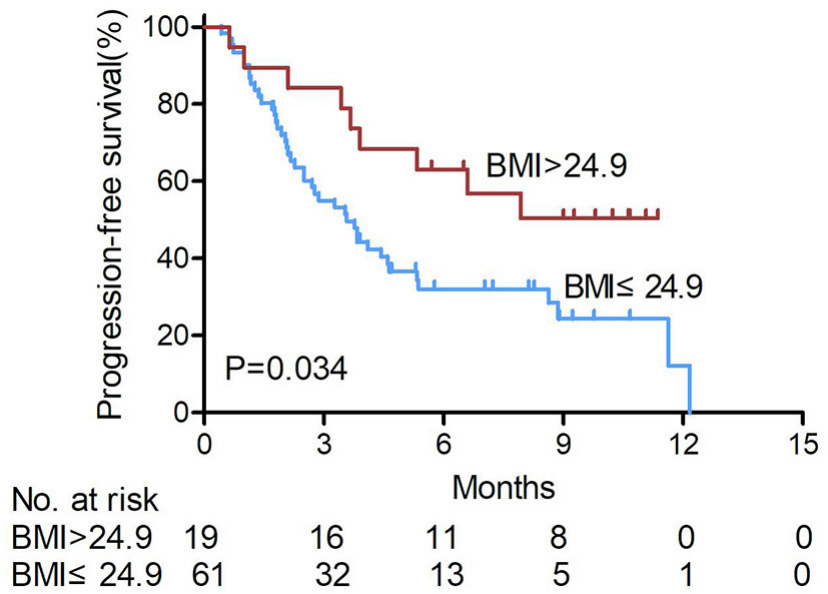
Kaplan-Meier survival curves of hepatocellular carcinoma patients treated with anti- PD-1 were analyzed in those without immune- related adverse events according to BMI levels. The PFS of overweight patients with irAEs was significantly longer than that of non-overweight patients (log-rank test). BMI, body mass index. non-over weight BMI ≤ 24.9, over weight BMI > 24.9.

In irAEs- patients (*n* = 80, 79.2%), the mean PFS of overweight patients (23.8%, 19/80) was 7.72 months, and that of non-overweight patients (76.2%, 61/80) was 5.31 months. The PFS of overweight patients with irAEs was significantly longer than that of non-overweight patients (*P* = 0.034, shown in Figure 2).

**Figure 3:**
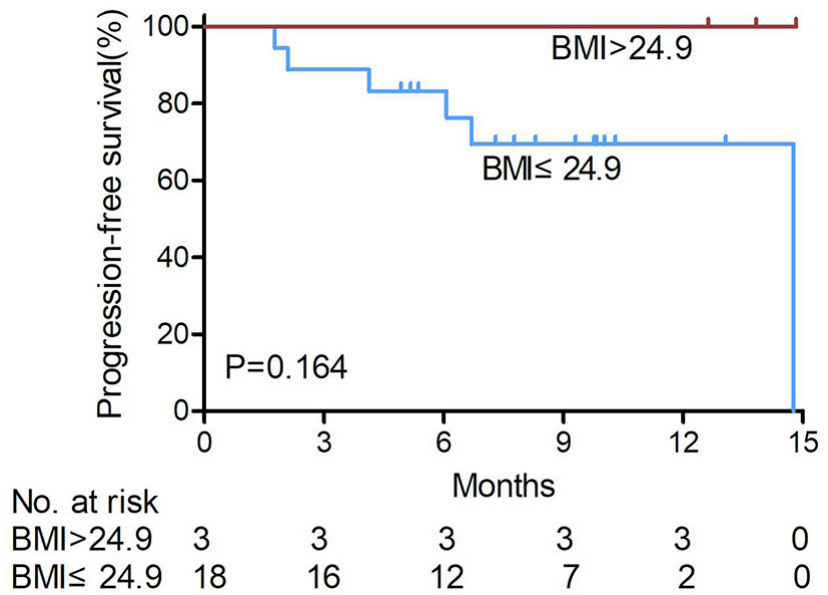
Kaplan-Meier survival curves of hepatocellular carcinoma patients treated with anti- PD-1 were analyzed in those with immune- related adverse events according to BMI levels. Among the irAEs+ patients, overweight patients had longer PFS than non-overweight patients, and no disease progression was observed in the three overweight patients after anti-PD-1 treatment (log-rank test). BMI, body mass index. non-over weight BMI ≤ 24.9, over weight BMI > 24.9.

Among the irAEs+ patients, overweight patients (14.3%, 3/21) also had longer PFS than non-overweight patients (85.7%, 18/21) (*P* = 0.164, shown in Figure 3), and no disease progression was observed in the three overweight patients after anti-PD-1 treatment.

## Discussion/conclusion

Anti-PD-1 antibodies have shown remarkable curative effects in the clinical treatment of patients with HCC, but individual differences influencing their efficacy are obvious. Therefore, it is important to develop and evaluate the efficacy prediction and prognostic indicators of anti-PD-1 antibodies in patients with HCC. BMI, as a simple and stable indicator, can effectively predict the response of patients with HCC receiving anti-PD-1 antibody treatment, which is helpful for the clinical management of such patients. In this study, we demonstrated a strong association between BMI and the efficacy of anti-PD-1 antibodies in patients with HCC. Patients with a BMI>24.9 had better clinical outcomes than those with a BMI ≤ 24.9.

BMI is one of the criteria used to measure nutritional status. High BMI is often considered a risk factor for cancer and is associated with cancer progression (15–17). However, recent studies have suggested that higher BMI is associated with better outcomes in patients receiving immunotherapy for metastatic melanoma and non-small cell lung cancer (18, 19). These results are consistent with our findings.

The immune checkpoint molecule PD-1 mainly downregulates the immune system response to human cells by inhibiting the inflammatory activities of T cells. Malignant tumors lead to the increased expression of checkpoint proteins, thus preventing T cells from attacking cancer cells and enabling immune escape. However, ICIs block these proteins, enabling T cells to continue tracking and killing cancer cells (20). It has been suggested that obesity increases the expression of checkpoint proteins. In obese people, owing to the accumulation of fat cells, the human body secretes leptin to inhibit the synthesis of fat cells to avoid excessive obesity. Leptin can activate STAT3-dependent signaling pathways, promote oxidative phosphorylation of CD8+ T cells, and induce increased PD-1 expression and inhibition of T cell proliferation (21). In one study, the expression of PD-1 protein in the T cells of obese mice was significantly higher than that in non-obese mice; accordingly, the anti-PD-1 effect of obese mice with tumors was better than that of non-obese mice, and the survival time was longer (22).

Additionally, many studies have suggested that anti-PD-1 efficacy is associated with irAEs (23). Although the mechanism underlying the occurrence of irAEs is not clear, irAEs have been shown to improve the efficacy of anti-PD-1 antibodies. The occurrence of irAEs has also been reported in liver cancer, especially in patients with rashes and with a significantly longer median PFS (24). In obese patients, the rate of inflammatory response increases and the probability of irAEs also increases (25, 26). This is because macrophages in the fat tissue of obese people are more M1 type, which mainly secrete inflammatory cytokines, while macrophages in the fat tissue of non-obese people are M2 type, which mainly secrete anti-inflammatory cytokines. Pro-inflammatory Th1 and CD8 + T cells gradually outnumber anti-inflammatory Th2 and Treg cells in obese individuals (27, 28). Therefore, obesity is often associated with low levels of chronic inflammation in adipose tissue. Our study also showed that BMI was a good stratification factor in patients without irAEs, and patients without irAEs but with a higher BMI had better prognosis. Although BMI had poor predictive efficacy for the PFS of liver cancer cases with irAEs, failing to reach the set significant difference, survival analysis showed that overweight patients who developed irAEs had longer PFS in our study cohort.

Some evidence shows that BMI is correlated with the prognosis of targeted and immunotherapy for cancer (11, 19), but no studies have reported the prognostic role of BMI in anti-PD-1 therapy for HCC patients. In our study, BMI was shown to be an independent prognostic factor for HCC patients receiving anti-PD-1 treatment. A higher BMI is significantly correlated with longer PFS, and BMI can be used as a prospective stratification factor in the clinical trials of patients with HCC. BMI can also be used as a prognostic marker for liver cancer patients treated with anti-PD-1 and can assist in the clinical prediction of patient prognosis.

However, this study has some limitations. Due to the limited data on HCC patients receiving anti-PD-1 antibody treatment, we only studied 101 cases in our center, thereby lacking a multicentre case cohort for verification. In future studies, multicentre case cohort verification can be carried out jointly, and mechanistic experiments can be used to further clarify the relationship between BMI and anti-PD-1 efficacy.

In conclusion, a higher BMI predicts a better prognosis among HCC patients treated with anti-PD-1 therapy; BMI was a good stratification factor for PFS in the cohort of HCC patients without irAEs. BMI combined with irAEs in patients with HCC can be a useful tool for predicting anti-PD-1 efficacy.

## Data availability statement

The authenticity of this article has been validated by uploading the key raw data onto the Research Data Deposit public platform (www.researchdata.org.cn), with the approval RDD number as RDDA2022245274.

## Ethics statement

The studies involving human participants were reviewed and approved by the Institutional Review Board of Sun Yat-sen University Cancer Center (2020-FXY-166- liver surgery). The patients/participants provided their written informed consent to participate in this study.

## Author contributions

JwC, WC, and LZ designed this study. LL collected and analyzed the data. JrC wrote the manuscript. CQ, GA, and FD assisted in analyzing the data. JwC and MC revised the manuscript. All authors contributed to the article and approved the submitted version.

## Funding

This study was supported by the Youth Foundation of National Natural Science Foundation of China (81902420), Youth Innovation Promotion Program of Sun Yat-sen University Cancer Center (QNYCPY22), Guangdong Esophageal Cancer Institute Science and Technology Program (No. Q201903), and Science and Technology Program of Guangzhou, China (202102080436).

## Conflict of interest

The authors declare that the research was conducted in the absence of any commercial or financial relationships that could be construed as a potential conflict of interest.

## Publisher's note

All claims expressed in this article are solely those of the authors and do not necessarily represent those of their affiliated organizations, or those of the publisher, the editors and the reviewers. Any product that may be evaluated in this article, or claim that may be made by its manufacturer, is not guaranteed or endorsed by the publisher.

## Abbreviations

AFP: alpha- fetoprotein
AST: aspartate aminotransferase
BMI: body mass index
CR: complete response
CRE: creatinine
DCR: disease control rate
HCC: hepatocellular carcinoma
ICIs: Immunocheckpoint inhibitors
irAEs: immune-related adverse events
ORR: overall response rate
PD: progressive disease
PD-1: Programmed cell death receptor-1
PFS: progression-free survival
PR: partial response
SD: stable disease.
